# Assessment of Root Canal Enlargement Using Mtwo and BioRace Rotary Files

**DOI:** 10.1155/2015/859693

**Published:** 2015-03-23

**Authors:** Enzo Cumbo, Riccardo Russo, Giuseppe Gallina

**Affiliations:** ^1^Sezione di Scienze Stomatologiche, Insegnamento di Endodonzia, Università di Palermo, Via del Vespro 123, 90123 Palermo, Italy; ^2^Policlinico Paolo Giaccone, Sezione Scienze Stomatologiche G. Messina, Università di Palermo, Via del Vespro 129, 90100 Palermo, Italy; ^3^Sezione di Scienze Stomatologiche, Università di Palermo, Via del Vespro 123, 90123 Palermo, Italy; ^4^Sezione di Scienze Stomatologiche, Insegnamento di Odontoiatria Conservatrice, Università di Palermo, Via del Vespro 123, 90123 Palermo, Italy

## Abstract

*Objective*. To evaluate root canal enlargement following mechanical shaping using 2 nickel titanium rotary systems. *Material and Methods*. Forty single-rooted teeth were immersed in resin and sectioned perpendicular to the long axis at 4, 8, and 12 mm from the apex. Digital capture of sections was performed before and after canal instrumentation using Mtwo and BioRace instruments. The area increase of endodontic space was calculated by subtraction. *Results*. The use of both instruments has allowed the removal of great amounts of dentin from the canal walls, even when the endodontic morphology is characterized by awkwardness to reach recesses. *Conclusions*. Both procedures seem to be valid and no differences were found between Mtwo and BioRaCe considering the amount of dentin removed at different distances from the apex.

## 1. Introduction

In the last decade, the introduction of new technologies such as nickel-titanium (NiTi) instruments and new canal filling systems have allowed the dentist, together with the use of microscopes, to set even more effective therapeutic protocols [1, 2]. The introduction of these instruments has enabled root canal instrumentation to be faster while remaining respectful of the original root canal anatomy [3].

This can be further possible thanks to the file design [4, 5] and the crown-down approach [2, 6]. Several NiTi instruments are available on the market. Besides the metal type, instrument geometry is the major factor that influences the behavior of instruments towards torque stresses, fracture strength, rotation speed, and operator sensitivity [7].

The first generation NiTi instruments had poor shaping ability (neutral cutting angle) and nowadays a lot of instruments with great shaping ability (positive cutting angle) are available on the market. This characteristic has inevitably modified their use; in fact, while the first generation instruments, not very sharp, required longer appointments for the patients the latest instruments, with greater shaping ability, allow the dentist to perform faster procedures and shorter appointments [8–11].

The aim of this study is to compare dentin removal during shaping with 2 different nickel-titanium systems by measuring the cross-sectional area of the root canals before and after instrumentation.

## 2. Materials and Methods

Forty human teeth single-rooted, without restorations, with intact crowns, and fully formed apices, extracted for orthodontic and/or periodontal reasons, were selected. All teeth were cleaned in 5% NaOCl solution for 24 h, carefully cleaned of periodontal tissue and calculus, washed under running water, dried, and stored in 10% formalin solution. All specimens had a root canal with a curvature angle lower than 20° that was evaluated using the Schneider technique [8, 12] by two radiographs (mesial-distal and buccal-lingual). Roots with resorptions, fractures, and open apices were excluded and every tooth had its crown removed at the cement-enamel junction (mesial side). The working length of the canals was determined by observing a file number 10 protruding through the apical foramen and subtracting 0.5 mm from the recorded length. An experimental model was made, capable to standardize the position of every sample, using the Bramante modified technique [13].

**Figure 1:**
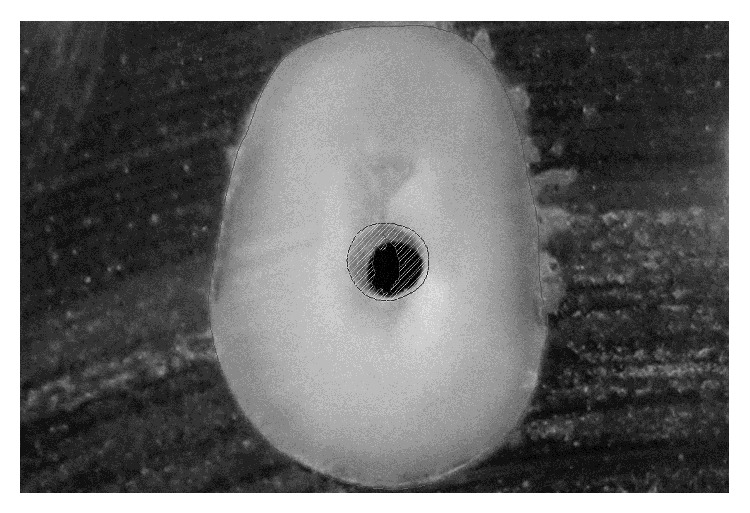
Example of area before and after reaming.

All specimens were immersed in self-curing transparent resin (Viapal uP 0004/64; Vianova Resin, Hamburg, Germany), in order to create resin blocks which were cut to obtain sections containing resin and a root portion. For each specimen, 3 sections perpendicular to the longer axis were created at 4, 8, and 12 mm from the apical foramen. Digital captures of all sections (coronal view) were recorded before reassembling them using a repositioning device to file the canals [14] (Figure 1). All the resin blocks were randomly divided into 2 groups, A and B, of 20 samples each.

Group A was instrumented with Mtwo (Sweden & Martina, Padova, Italy); group B was instrumented with BioRace (FKG Dentaire, La Chaux de Fonds, Switzerland).

For both groups, every NiTi instrument was used just for 8 seconds (which is less than the suggested working time from the manufactures) and only for 5 canals so any instrument was used only for 40 seconds in order to preserve the sharpening; irrigation was carried out with EDTA-based Glyde chelating solution (Maillefer, Ballaigues, Switzerland), alternating it with NaClO Niclor 5 (Ogna, Muggio, Italy) to facilitate the progression of the instrument inside the canal, to reduce the torsional stress and minimize the usury on the blades [3].

**Figure 2:**
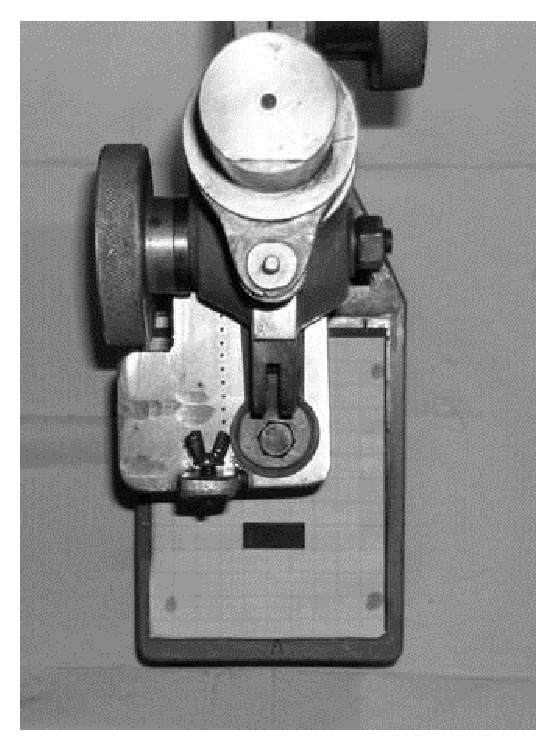
Stand to maintain a digital camera and the sections in a repeatable position.

For any digital capture, a stand (Figure 2) was created to maintain a digital camera (Coolpix 5400, Nikon, Japan) and the sections in a repeatable position, to permit pre- and postpreparation image comparison through superimposition. Every image was digitalized before reaming in order to store the original morphology of the endodontic section (coronal view). Root canal shaping was always performed by the same operator. The clinical protocols were carried out using the following sequences. 

*Group A.* Mtwo: 10/.04, 15/.05, 20/.06, 25/.06. The simultaneous technique without any early coronal enlargement (total 32 seconds). 

*Group B.* BioRace: 25/.08, 15/.05, 25/.04, 25/.06 (total 32 seconds) (crown-down technique) [6, 15–17].

It is remarkable that, in this study, any instrument (except BR0) was taken at the working length, with light apical pressure, and used just for 8 seconds; all the tested instruments were used with lateral pressure (brushing mode) to obtain a circumferential cut.

After shaping, all specimens were disassembled and the sections were repositioned on the stand for another capture. Digital image analysis was carried out with AutoCAD graphic software (Autodesk Inc, USA) [4], which permitted the highlighting of endodontic space profiles before and after reaming; red color (before shaping) and blue color (after shaping) were used.

Statistical data analysis was carried out with Statistica software v.6.1 (StatSoft Italia s.r.l). The significance of the differences in pre- and postshaping areas at the 3 sections was evaluated in both groups with the Wilcoxon test; the significance level was fixed at *P* < 0.05.

## 3. Results

During simulated clinical use no instrument had intracanal fracture, while some files of both groups showed small visible signs of plastic deformation, especially close to the tip; no transportation of the root canal or strip perforation occurred.

The operator, in the evaluation stage, was blinded to the type of file and all results are summarized in Table 1.

The increase in the postpreparation endodontic space area in group A (Mtwo) was statistically significant in all 3 sections (coronal: *P* = 0.000089, middle: *P* = 0.000022, and apical: *P* = 0.000022).

In group B (BioRace) pre- and postpreparation differences were statistically significant in all 3 sections (coronal: *P* = 0.00002, middle: *P* = 0.000022, and apical: *P* = 0.000089).

The comparison between the two groups of samples at the coronal, middle, and apical sections, carried out with the Mann-Whitney *U* test, did not show statistically significant differences between the two different types of rotary instruments (coronal sections: *P* = 0.7643, middle sections: *P* = 0.1202, and apical sections: *P* = 0.1460).

## 4. Discussion

Within the limits of an “in vitro” study, the Bramante technique [13] (modified by Kuttler et al. [14]) offers a method that is relatively simple and economical and provides useful information about the action of instruments in the canal space. An alternative method of assessing root canal instrumentation techniques is the microcomputer tomography that is more expensive and requires well-trained operators in order to obtain valid results.

This study evaluated two different procedures based on NiTi rotary instruments that were used for just 32 seconds inside the canal and without preflaring with Gates-Glidden or Largo burs.

In ideal conditions, both files seem to create rapidly a round shape regardless of the initial root canal's morphology. The analysis of the results showed the shaping ability for both types of instruments that permitted proper dentin removal from the canal walls; and pre- and postpreparation differences were statistically significant in all 3 sections.

The Mtwo removed smaller amounts of dentine compared to BioRace at the coronal sections. NiTi Mtwo instruments (simultaneous technique) do not remove indiscriminately coronal root dentine with an early coronal enlargement, but rather progressively eliminate dentine at the orifice through a selective coronal enlargement [17].

Given the cutting ability of the rotary instruments tested, few seconds were sufficient to ensure a proper shaping reducing, at the same time, stresses in the NiTi alloy. Even if the canal preparation shape became dictated more by anatomy than by differences in instrumentation method [18] both types of instruments tested showed a similar tendency to modify the canal walls. Even though the use of Mtwo files compared with canal preparation with K3 or RaCe instruments showed a less production of debris, as described by Schäfer et al. [19], within the parameters of this study, the statistical analysis did not reveal a significant difference between the Mtwo group and the BioRace group considering the quantity of dentin removed at all different levels. The lack of significance between the two groups may be a consequence of the high degree of similarity between them; a variety of root canal anatomy within the groups may have produced a relatively high dispersion of the data.

Both rotary instruments tested were also used with a brushing motion which may have influenced the final shape of all canals more than the differences (shaping ability) between Mtwo and BioRace.

Considering our data, simple procedures and sharp rotary instruments, such as Mtwo and BioRace tested in this study, may allow the dentist, in few minutes, to obtain an efficient enlargement of the root canals.

## Figures and Tables

**Table 1:** Results.

Sections	Group A		Group B	
	Area increase	Mean	Area increase	Mean
	mm^2^	mm^2^	mm^2^	mm^2^
Coronal	0.15 to 0.8480	0.4964 ± 0.2072	0.1901 to 1.1242	0.6571 ± 0.2323
Middle	0.0864 to 0.6223	0.3233 ± 0.1536	0.0532 to 0.6012	0.3272 ± 0.1432
Apical	0.0078 to 0.5499	0.1930 ± 0.1565	0.0052 to 0.4832	0.1431 ± 0.1235
